# Salermide down-regulates sirtuin proteins to induce human cancer cell apoptosis

**DOI:** 10.1186/1471-2210-11-S2-A49

**Published:** 2011-09-05

**Authors:** Asha Leisser, Michael Wolzt, Angela Storka

**Affiliations:** 1 Department of Clinical Pharmacology, Medical University of Vienna, 1090 Vienna, Austria

## Background

The NAD^+^-dependent family of sirtuin proteins (SIRT1–7), is involved in cell apoptosis and senescence. Salermide is a potent inhibitor of SIRT1 and SIRT2 and can induce tumor-specific cell death in selected human cell lines. In this study we investigated salermide’s apoptotic effect in a wide range of other human cancer cell lines and its antiproliferative potential in combination with cisplatin.

## Methods

Seven different cancer cell lines (SKOV-3, MKN45, MKN28, N87, FaDu, NuLi1, Jurkat) were treated with salermide (1 µM – 0.1 nM) for 24, 48, and 72 hours and assessed for cell viability. Three cell lines (SKOV-3, N87, Jurkat) were selected for combination therapy with salermide and cisplatin (30 µM). In order to characterize salermide’s proapoptotic pathway SIRT1, SIRT2, pAKt, p53, acetyl-p53 and Nampt (nicotinamide phosphoribosyltransferase) were determined in SKOV-3 and Jurkat cells by Western blotting.

## Results

Salermide yielded greater dose-dependent apoptotic effects in Jurkat, SKOV-3 and N87 cells than in the other cell lines, with most potent effect after 48 h of incubation. The anti-proliferative activity was associated with a G_0_-G_1_ cell cycle arrest. SIRT1 and SIRT2 protein were down-regulated after 48 h and 72 h. This was accompanied by a down-regulation of pAKT, p53 and Nampt. Acetyl-p53 levels were not consistent across cell types. Cisplatin exerted synergistic effects with salermide in all cell lines and reduced cell viability up to 50%.

## Conclusions

Salermide-induced apoptosis is cell line-dependent and more effective in slow-proliferating (SKOV-3) and hematologic (Jurkat) cancer cells. The synergism with cisplatin implies a potentiating effect of this sirtuin inhibitor as add-on in clinical cancer therapy.

